# Differentiation in drought tolerance mirrors the geographic distributions of alpine plants on the Qinghai-Tibet Plateau and adjacent highlands

**DOI:** 10.1038/srep42466

**Published:** 2017-02-14

**Authors:** Li-Hua Meng, Jie Yang, Wen Guo, Bin Tian, Guang-Jie Chen, Yong-Ping Yang, Yuan-Wen Duan

**Affiliations:** 1 School of Life Sciences, Key Laboratory of Yunnan for Biomass Energy and Biotechnology of Environment, Yunnan Normal University, Kunming 650092, P. R. China; 2 Kunming Institute of Botany, Chinese Academy of Sciences, Kunming 650201, P. R. China; 3 Key Laboratory of Plateau Lake Ecology and Global Change, School of Tourism and Geography, Yunnan Normal University, Kunming 650500, P. R. China

## Abstract

Climatic tolerance, especially drought tolerance, is one of the major factors shaping the geographic distributions of plant species. Thus, the general decline in rainfall from the Himalaya-Hengduan Mountains (HHM) to the inner Qinghai-Tibet Plateau (QTP) might account for the significant differences in species distributions and richness between the two regions. To test this hypothesis, we conducted a water stress experiment using four *Anisodus* species (*A. tanguticus, A. luridus, A. carniolicoides* and *A. acutangulus*), which were treated with different levels of water stress in a glasshouse, and examined their differences in physiological responses. The results suggest that *A. tanguticus*, which inhabits the inner QTP, generally has higher fitness under severe water stress than the other species based on its high root:shoot ratio, long-term water use efficiency and photosynthetic rate, indicating that it possesses a genetically based drought tolerance mechanism. Our results suggest that plant species inhabiting the inner QTP may be more drought tolerant than those inhabiting the HHM regions. This provides a new example supporting the hypothesis that climatic tolerance plays a major role in shaping plant distributions on the QTP and its adjacent highlands and presents new insights into the patterns of geographic distribution and diversity of the plants inhabiting these areas.

One of the fundamental goals for ecologists and evolutionary biologists is to explain the patterns and mechanisms involved in species geographic distributions and abundance^1^. This could aid in the understanding of numerous phenomena, including the formation of biodiversity in a given region^2^. Efforts to achieve this goal can be facilitated by elucidating the factors and mechanisms that govern the remarkable variation in geographical distributions within congeneric taxa. Among the potential mechanisms proposed^3^,^4^, climatic tolerance is considered to play an important role in shaping plant distributions^5^,^6^. *Inter alia*¸ it is generally hypothesized that only plants that can tolerate the most extreme climatic conditions in a given region will be able to colonize, survive, and reproduce where these conditions occur, as they have competitive advantages in such areas^7^. Drought is one of the most important abiotic environmental factors^8^,^9^,^10^ affecting plant growth and reproduction^11^,^12^ and distribution^13^. Accordingly, sensitivity to drought is considered to strongly influence the geographic distributions of both species and communities^13^,^14^,^15^. However, the validity of this hypothesis has been rarely tested experimentally for alpine species inhabiting the Qinghai-Tibet Plateau (QTP) and its adjacent highlands, the QTP being the largest and highest plateau in the world^16^ and considered to be one of the world’s biodiversity hotspots^17^.

In general, the origins of various contemporary plant species on the QTP have been identified in the past decade^18^,^19^, but many issues still require clarification. Notably, there are more than 12,000 species of flowering plants in the Himalaya-Hengduan Mountains (HHM) regions and the inner QTP in total, but only ca. 4000 are found on the inner QTP. The reasons for this are not fully understood but can be strongly associated with the uplift of the QTP and the resulting major climatic changes in the region. The climate of the inner QTP gradually became drier, colder and windier, resulting in the development of glaciers and deserts and the replacement of forests by grasslands^20^. This is considered to have occurred mainly due to the development of Asian monsoons^21^ and an associated decline in summer rainfall from the outer to the inner QTP^22^,^23^ (Fig. 1A). Accordingly, the QTP and its adjacent highlands, including the HHM, provide a natural system to test the effects of drought gradients on the geographic distribution of plant species. We may predict, for instance, that only plant species that can tolerate dry conditions could have colonized the platform of the QTP. Experimental tests of this and similar predictions could help to understand the pressures that have shaped the modern distribution and diversity of plant species on the QTP and the adjacent highlands.

*Anisodus*, a small genus of Solanaceae, includes only four species^24^. *Anisodus tanguticus* occupies the inner QTP, but the other three species (*A. luridus, A. acutangulus* and *A. carniolicoides*) are restricted to the HHM regions and are never found on the inner QTP^25^ (Fig. 1B). Therefore, *Anisodus* may be a useful model for studying the relationships between interspecific differences in drought tolerance and the geographic distributions of plants on the QTP and HHM. Specifically, we hypothesized that *A. tanguticus* is more tolerant to drought conditions than its three other congeneric *Anisodus* species. Thus, we examined the responses of *A. tanguticus* and its congeneric species to changes in soil water content to test the hypothesis that only plants with higher drought tolerance could colonize and survive on the inner QTP as a contribution to broaden efforts to enhance the understanding of the current geographic distribution of plant species and the formation of biodiversity on the inner QTP and its adjacent highlands.

## Results

### Plant biomass and water use efficiency

With the decrease in soil water content in the pot experiment, both the aboveground biomass (AB) and belowground biomass (BB) of all four *Anisodus* species decreased significantly, with the exception of BB for *A. tanguticus*, leading to reductions in the total dry mass (TB; Table 1). Furthermore, the AB, BB and TB values were smallest for *A. tanguticus* (Table 1), and the three variables were significantly affected by species, water treatment and their interaction (Table S1). However, the root:shoot ratio (R:S ratio) of *A. tanguticus* increased significantly with a reduction in water availability, while no clear water-related trend in the R:S ratios of the other three species was detected (Fig. 2). This indicates that the biomass allocation responses to changes in soil water content differ among the four species (Table S1).

Similarly, the long-term water use efficiency (*WUE*_L_) of *A. tanguticus* increased significantly with a reduction in soil water content, but no significant between-treatment differences in this variable were detected in the other three *Anisodus* species (Table 1). This corroborates the differences in water use efficiency among the four *Anisodus* species (Table 1). With increasing water stress, significant decreases in the transpiration rate (*E*) were observed in all four species, but the *WUE*_i_ (the *A*_max_ to *E* ratio) increased significantly in all species (Table 1). There were also significant between-species differences in both of these traits under each treatment (Table 1, S1). The carbon isotope composition (δ^13^C), an indicator of the integrated water use efficiency of plant species^26^,^27^, significantly increased with increases in water stress in all four species, but it was generally lowest in *A. tanguticus* leaves and significantly lower than in the leaves of the other species under the moderate and low water stress treatments (Table 1, S1).

### Leaf physiological traits and chlorophyll fluorescence

Both the maximum photosynthetic rate (*A*_max_) and stomatal conductance (*g*) increased significantly with a decrease in water stress and significantly differed among species under each treatment (Table 1, S1). More specifically, *A. tanguticus* had the highest values of these variables under the high water stress treatment, but *A. luridus* had higher values under the moderate and low water stress treatments (Table 1).

The maximum quantum yield of PS II (*F*_v_/*F*_m_) was significantly lower under the high water stress treatment than under the moderate and low water stress treatments in *A. tanguticus* and *A. acutangulus*, but no significant between-treatment differences in this variable were detected in *A. luridus* and *A. carniolicoides* (Table 1). Significant differences in this respect were observed among species under each of the water treatments (Table 1, S1).

### Nitrogen use efficiency (NUE) and carbon content

The nitrogen use efficiency (NUE) of *A. luridus* and *A. carniolicoides* seedlings was significantly lower under high water stress than under the other treatments, but no significant between-treatment differences in NUE were detected in *A. tanguticus* and *A. acutangulus* (Table 1). Thus, the NUE was only significantly affected by the water treatment (Table S1). The carbon content (%) was affected by both treatments and species (Table S1), but a significant difference among water treatments was only found in *A. tanguticus* (Table 1). The carbon content was generally higher in *A. tanguticus* than in the other three *Anisodus* species, but the difference was not significant across all treatments (Table 1).

## Discussion

### Ecophysiological responses of four Anisodus species to drought stress

**Figure 1:**
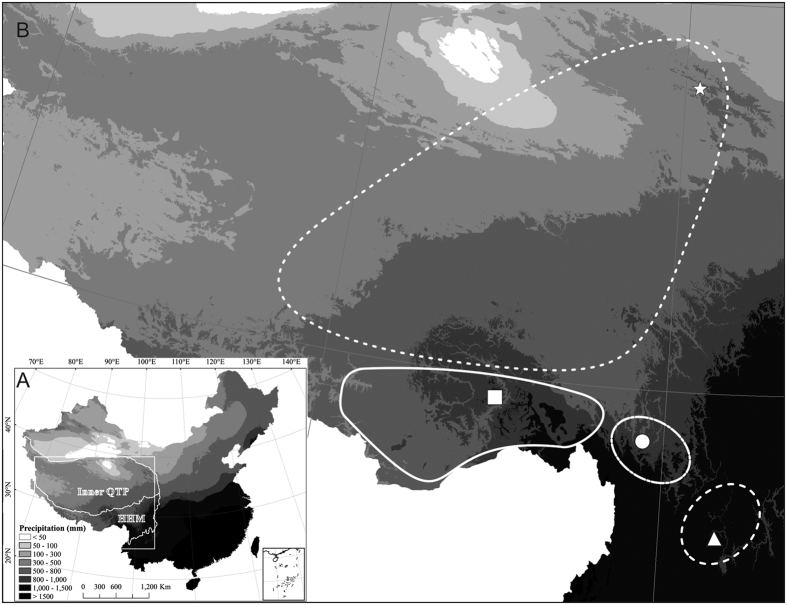
(**A**) The general scope of the inner QTP and HHM and the distribution of average rainfall from 1990 to 2010 during the growing season (May to September) in China and the study regions, which were generated by Y.-W. D. in ArcGIS (ver. 10.2) (https://www.arcgis.com/features/) based on public data from the China Meteorological Administration (http://data.cma.cn/). (**B**) Ranges of *A. tanguticus* (dotted line), *A. luridus* (line), *A. carniolicoides* (dotted and dashed line) and *A. acutangulus* (dashed line). The star, square, dot and triangle indicate sources of seeds for the glasshouse water stress experiment.

**Figure 2:**
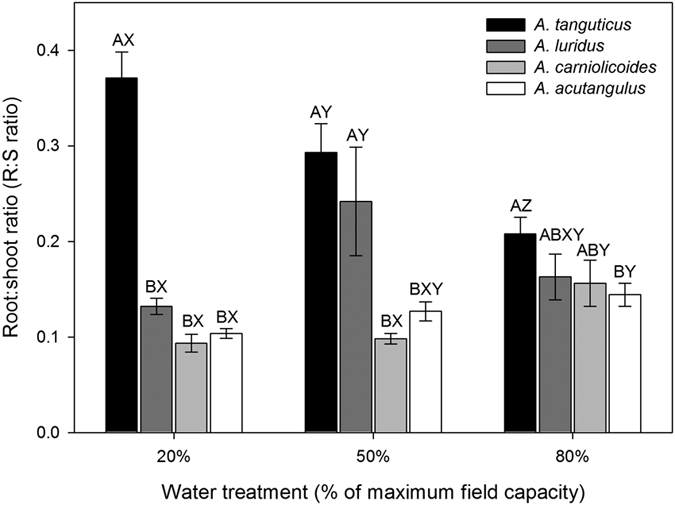
Root:shoot ratio (R:S ratio) of seedlings of *A. tanguticus* (black bars), *A. luridus* (dark grey bars), *A. carniolicoides* (grey bars) and *A. acutangulus* (open bars) under different soil water conditions. Presented data are means + SE. Values with different letters (X, Y, Z) indicate a significant difference (*P* < 0.05) between water treatments, and those with different letters (**A**, B, C) indicate a significant difference (*P* < 0.05) between species, based on one-way ANOVA.

Water deficits can limit plant growth, reproduction and distribution, and thus various mechanisms have evolved in plants inhabiting water-limited regions to enhance drought tolerance by adjusting their physiological and morphological traits^28^,^29^. *Anisodus tanguticus* occupies the inner QTP, while the other three *Anisodus* species are restricted to the HHM regions, and rainfall in the growing season (May-September) on the inner QTP is significantly lower than in the HHM regions (Fig. 1A). Therefore, *A. tanguticus* might be expected to be more tolerant to drought stress than the other three *Anisodus* species. Our results show that increases in water stress reduce the growth of all four species, which could be manifested by the lower accumulation of aboveground, belowground and total biomass (Table 1). A reduced increase in biomass is a common response of plant species to drought stress because drought stress can facilitate stomatal closure and then reduce the photosynthetic rate^30^, which could be found in our results. Furthermore, since plants must enhance their ability to acquire water to cope with water deficits under drought conditions, the relative allocation of dry matter between shoots and roots would also change under different levels of water stress^31^,^32^. Accordingly, an increase in biomass allocation to root growth is generally observed in plants under water stress^28^,^30^. In our experiments, the R:S ratio of *A. tanguticus* seedlings significantly increased with an increase in water stress, and the R:S ratio of *A. tanguticus* was generally higher than that of the other three species under each water treatment (Fig. 2). Collectively, compared to the other three *Anisodus* species, resource allocation to root growth is enhanced in *A. tanguticus* even under no water stress (80% FC), indicating that *A. tanguticus* has evolved genetically based drought tolerance mechanisms to cope with water deficits.

As we have mentioned above, soil water availability can also affect stomatal conductance (*g*) and maximal photosynthetic rates (*A*_max_)^33^. In our experiments, *A*_max_ was ca. 6.7%, 44.9%, 15.6% and 13.8% lower in highly water-stressed seedlings than in the moderately stressed seedlings of *A. tanguticus, A. luridus, A. carniolicoides* and *A. acutangulus*, respectively. Similarly, there were ca. 37.5%, 78.3%, 21.9% and 19.2% reductions, respectively, in stomatal conductance. Similar trends were also detected in transpiration rates (*E*) (Table 1), which could result from changes in stomatal conductance. These results support the hypothesis that stomatal conductance is more sensitive to drought than to photosynthesis rates^34^. Consequently, the *WUE*_i_ (*A*_max_/*E*) values of highly water-stressed seedlings were 30.3%, 65.9%, 17.0% and 4.8% higher than those of the moderately water-stressed seedlings of *A. tanguticus, A. luridus, A. carniolicoides* and *A. acutangulus*, respectively. The maximum quantum yield of PSII (*F*_v/_*F*_m_), which indicates plant photosynthetic activity^35^, also decreased with increases in water stress. Among the highly water-stressed seedlings, the photosynthetic rate of *A. tanguticus* seedlings was the highest, but the maximum quantum yield of PSII was generally the lowest, which might partially explain the weaker observed growth of *A. tanguticus*.

Water stress usually increases the water use efficiency of plant species^36^, which was also observed in *A. tanguticus*, as the *WUE*_L_ of this species was significantly higher than that of the other three *Anisodus* species under the severe and moderate water stress treatments (Table 1), suggesting that *A. tanguticus*, with higher water use efficiency, should have a greater ability to survive drought stress than those with lower water use efficiency^37^. In contrast, our δ^13^C measurements show that water use efficiency increases slightly with an increase in water stress in all four *Anisodus* species, as suggested by previous studies^30^,^38^. As δ^13^C and *WUE* are both affected by intercellular to ambient CO_2_ partial pressures (*P*i/*P*a), stomatal conductance (*g*) and the photosynthetic rate (*A*), they are usually positively related^39^,^40^. However, in our results, *A. tanguticus* had a higher *WUE*_L_ but a lower δ^13^C than the other three *Anisodus* species (Table 1). The higher photosynthetic capacity of *A. tanguticus* may reduce *P*i/*P*a, leading to the low value of δ^13^C in this species under severe water stress^41^. In addition, a high δ^13^C is generally correlated with great biomass accumulation in many species^42^,^43^, and thus the low δ^13^C for *A. tanguticus* may reflect the different growth rates during desiccation in comparison with the other three *Anisodus* species.

Decreasing soil water availability negatively affects nutrient input, decomposition, and mineralization, all of which should decrease plant-available nutrients^44^,^45^. In our experiment, the NUE of *A. luridus* and *A. carniolicoides* decreased with a reduction in water supply (Table 1), implying that water stress strongly limits N uptake^46^. The low NUE of *A. tanguticus* suggests that a high *WUE*_L_ might induce a low NUE because it is thought that species native to dry sites tend to conserve water and pay for that conservation with low returns on leaf nitrogen^47^. The carbon contents of these four *Anisodus* species decreased from 80% FC to 20% FC (Table 1), indicating that water stress might play a primary limiting role in C accumulation^48^, but a slightly higher carbon content was found in seedlings of *A. tanguticus* than the other three species under each water treatment. All these results, together with the prioritization of root allocation during plant growth, strongly indicate that *A. tanguticus* responds to water stress differently compared with its three congeners and could have higher ecological fitness than the other three species in arid environments^49^,^50^.

### Implications for the geographic distribution of plant species on the QTP and HHM

The QTP uplift and associated climatic effects, particularly the development of a monsoon-dominated weather pattern^51^, are believed to have triggered and facilitated plant speciation and diversification^18^,^19^. The HHM region of the eastern and southern parts of the QTP harbours exceptionally high species richness with high levels of endemism^52^,^53^. However, the reasons for such great differences in species richness between the inner QTP and the HHM region have been rarely explored. The mechanisms shaping plant distributions are complex, but climatic tolerance is considered to play a major role^5^,^6^. From the HHM to the inner QTP, both precipitation (Fig. 1A, from 500–800 mm to 100–300 mm) and mean air temperature (from up to 15 °C to ~7.5 °C) decline significantly during the growing seasons of plant species^23^. Decreases in rainfall from the HHM to the inner QTP have been considered to be important in the distribution patterns of vegetation for a long time^54^. Additionally, low temperatures could induce cold-induced drought stress, which might also limit the growth and reproduction of plants by inducing xylem cavitation^55^, a process similar to that induced by water deficit. Therefore, plant species inhabiting the inner QTP might be more tolerant of drought stress than those inhabiting the HHM region, which could be confirmed in our experiment because a generally high fitness is found in *A. tanguticus* inhabiting the inner QTP when this species is subjected to high water stress. Collectively, our results provide a new example supporting the hypothesis that climatic tolerance strongly influences the distribution of alpine plant species. Furthermore, although the mechanisms of drought tolerance might vary substantially among different plant species, our results provide novel insights into the patterns of geographic distributions and the development of plant biodiversity on the inner QTP and its adjacent highlands.

## Materials and Methods

### Plant materials

*Anisodus* is only represented by *A. tanguticus, A. luridus, A. carniolicoides* and *A. acutangulus*. Flowers of the four species are nodding and pollinated by bumblebees, ants and wasps^56^,^57^,^58^. The fruits of these species are capsules^25^.

### Glasshouse experiment

Seeds were collected from wild populations of *A. tanguticus* (in Qinghai, 37°39′N, 101°19′E, 3266 m a.s.l.), *A. luridus* (in Tibet, 29°46′N, 94°44′E, 3130 m a.s.l.), *A. carniolicoides* (in Yunnan, 28°24′N, 98°59′E, 3955 m a.s.l.), and *A. acutangulus* (in Yunnan, 27°00′N, 100°10′E, 3250 m a.s.l.) in 2013 (Fig. 1B) and stored in the China Germplasm Bank of Wild Species, Kunming Institute of Botany. In March 2014, these seeds were sown in a glasshouse at Yunnan Normal University, Kunming. In May 2014, pairs of seedlings of each species were transplanted into pots (height 24 cm; upper and lower diameters 21 and 17 cm, respectively). All pots were filled with a homogeneous mixture (0.55 kg) of peat and perlite (1:1 in volume), and a thin layer of perlite (ca. 2 cm) was placed on the soil surface of each pot to prevent the loss of water through evaporation. The maximum field capacity (FC, 1.59 kg) was determined gravimetrically.

The experiments started on July 7, 2014 (day t1) and were terminated on August 16, 2014 (day t2). In total, 96 pots containing each species were used in the subsequent experiments, in which 15 pots per species were used for initial biomass measurements (see detailed growth and water use section below). The 81 pots for each plant species were evenly divided into three groups, which were subjected to a high, moderate or low water stress treatment. Of the 27 pots in each treatment for each species, six pots were used to measure photosynthesis and chlorophyll fluorescence parameters, seven to analyse carbon isotope composition and leaf elements, and 14 to calculate increases in biomass (see below). The three water stress treatments involved watering to 20%, 50% and 80% of the maximum FC, and the soil water content was maintained by weighing each pot every 2 d. The water loss in each pot was recorded as previously described^59^.

### Growth and water use efficiency

Increases in biomass were examined during the experiment. The seedlings in 15 pots containing each species were harvested at the start of the experiment (t1), and the seedlings in 14 pots subjected to each treatment were harvested when the experiment ended (t2). The seedlings in each pot were pooled and separated into belowground (roots) and aboveground (stems and leaves) parts. We measured the dry weight of each part after drying at 80 °C for 48 h in an oven and then subtracted the dry biomass per pot at day t1 from that at day t2 during the experiment for the two parts for each species and treatment. The biomass accumulation per seedling was calculated after dividing by 2. The total dry mass (TDM) was calculated by summing the masses of the two parts, and the root to shoot (R:S) ratio was calculated by dividing the aboveground mass by the belowground mass^59^.

Using empty unsown pots as a control, we calculated the water loss for each pot by subtracting the weight of each pot before each watering event from that after watering; the pots were watered every 2 d during the experiment. Then, we calculated the amount of water transpired per pot per day by subtracting the mean water loss per empty pot per day (evaporation only) from the mean water loss per pot with plants per day (including both evaporation plus transpiration) for each species under each treatment. Using these data, we divided the total amount of water transpired per pot by two to obtain the total transpired water use (TWU). Lastly, we divided the TDM by the TWU to obtain the long-term water use efficiency (*WUE*_L_) per plant for each species and treatment.

### Leaf physiological traits and chlorophyll fluorescence

We measured *A*_max_, *g* and *E* parameters using a LI-COR 6400XT infrared gas analyser (IRGA; LI-COR Biosciences, Lincoln, NE, USA). All measurements were conducted using six seedlings of each species under each treatment from 11:00 to 14:00 h on August 17, 2014, which was a sunny day. Light was supplied by an LED lamp (LI-6400-02B, LI-COR Biosciences), and the light levels were kept at 1500 μmol·m^−2^ s^−1^, which is above the light saturation point for all four species according to the light response curves before the measurements. The external CO_2_ was provided by portable tanks containing a CO_2_/air mixture with a concentration of 400 μmol·mol^−1^. A CO_2_ injector (LI-6400-01, LI-COR Biosciences) was used to control the output of the tanks. The temperature was maintained at 25–28 °C, and the relative humidity was kept at 25–30%. Then, we divided *A*_max_ by *E* to calculate the instantaneous water use efficiency (*WUE*_i_).

We examined the chlorophyll fluorescence parameters of leaves that had been kept under dark conditions for 30 min in the morning between 05:00 and 06:00 h on August 17, 2014. We also measured the maximum quantum yield of photosystem II (PSII, *F*_v_/*F*_m_) with the formula (*F*_m_ − *F*_o_)/*F*_m_, using an LI-6400-40 leaf chamber fluorimeter (LI-COR Biosciences).

### Analysis of leaf elements and carbon isotope composition

In the laboratory, ca. 0.2 g of dry leaves from each plant were powdered with a TissueLyser (Retsch, Haan, Germany) and divided into two subsets. The nitrogen and carbon contents of one subset were analysed with a CHN analyser (Vario EL; Elementar, Hanau, Germany), and the reciprocal of the nitrogen content was employed as the nitrogen use efficiency (NUE). The other subset was combusted in an EA1108 elemental analyser (Carlo Erba, Milan, Italy) and analysed in a Finnigan Delta Plus isotope mass spectrometer (Thermo Finnigan MAT GmbH, Bremen, Germany) to examine the carbon isotope composition of the leaves (δ^13^C), which was calculated relative to the Pee Dee Belemnite (PDB) standard as the ratio (‰). The formula was δ^13^C = (*R*_sample_/*R*_standard_ − 1) × 1000, where *R*_sample_ and *R*_standard_ are the ratios of ^13^C:^12^C in the sample and the standard, respectively.

### Statistical analyses

A general linear model (Proc GLM) was employed to examine the effects of species, treatments and interactions on all measured variables in the pot experiment. One-way ANOVA was used to examine significant differences among species in each treatment or among treatments for each species.

## Additional Information

**How to cite this article:** Meng, L.-H. *et al*. Differentiation in drought tolerance mirrors the geographic distributions of alpine plants on the Qinghai-Tibet Plateau and adjacent highlands. *Sci. Rep.* **7**, 42466; doi: 10.1038/srep42466 (2017).

**Publisher's note:** Springer Nature remains neutral with regard to jurisdictional claims in published maps and institutional affiliations.

## Supplementary Material

Supplementary Table S1

## Figures and Tables

**Table 1:** Measured variables (means ± SE) of the four *Anisodus* species under each of the three soil water treatments: Low stress (80% of maximum field capacity, FC), Moderate stress (50% FC) and High stress (20% FC).

Variable and species	Water treatments (% of maximum FC)		
	High stress	Moderate stress	Low stress
	(20% FC)	(50% FC)	(80% FC)
Aboveground biomass (g)			
*A. tanguticus*	0.238 ± 0.017 A, X	0.339 ± 0.039 A, X	0.459 ± 0.053 A, Y
*A. luridus*	0.839 ± 0.092 B, X	1.306 ± 0.146 B, Y	1.380 ± 0.113 B, Y
*A. carniolicoides*	0.857 ± 0.111 B, X	1.185 ± 0.109 B, X	2.472 ± 0.293 C, Y
*A. acutangulus*	0.912 ± 0.075 B, X	1.436 ± 0.109 B, Y	2.187 ± 0.129 C, Z
Belowground biomass (g)			
*A. tanguticus*	0.088 ± 0.010 A, X	0.101 ± 0.016 A, X	0.097 ± 0.017 A, X
*A. luridus*	0.115 ± 0.018 A, X	0.325 ± 0.088 B, Y	0.229 ± 0.037 AC, XY
*A. carniolicoides*	0.087 ± 0.018 A, X	0.116 ± 0.013 A, X	0.422 ± 0.116 B, Y
*A. acutangulus*	0.098 ± 0.011 A, X	0.180 ± 0.018 A, Y	0.314 ± 0.029 BC, Z
Total biomass (g)			
*A. tanguticus*	0.326 ± 0.025 A, X	0.441 ± 0.053 A, XY	0.557 ± 0.067 A, Y
*A. luridus*	0.954 ± 0.108 B, X	1.632 ± 0.198 B, Y	1.609 ± 0.135 B, Y
*A. carniolicoides*	0.944 ± 0.127 B, X	1.301 ± 0.119 B, X	2.893 ± 0.387 C, Y
*A. acutangulus*	1.010 ± 0.086 B, X	1.616 ± 0.120 B, Y	2.500 ± 0.147 C, Z
Root:shoot ratio			
*A. tanguticus*	0.371 ± 0.027 A, X	0.293 ± 0.030 A, Y	0.208 ± 0.018 A, Z
*A. luridus*	0.132 ± 0.008 B, X	0.242 ± 0.057 A, Y	0.163 ± 0.024 AB, XY
*A. carniolicoides*	0.094 ± 0.009 B, X	0.098 ± 0.006 B, X	0.156 ± 0.024 AB, Y
*A. acutangulus*	0.104 ± 0.005 B, X	0.127 ± 0.010 B, XY	0.144 ± 0.012 B, Y
Long-term water use efficiency (g∙kg^−1^)			
*A. tanguticus*	5.710 ± 1.079 A, X	2.202 ± 0.522 A, Y	1.113 ± 0.147 A, Y
*A. luridus*	1.055 ± 0.128 B, X	1.192 ± 0.041 B, X	1.214 ± 0.074 AC, X
*A. carniolicoides*	2.117 ± 0.598 B, X	1.560 ± 0.124 B, X	2.838 ± 0.307 B X
*A. acutangulus*	1.217 ± 0.227 B, X	1.527 ± 0.069 B, X	1.629 ± 0.090 C, X
photosynthetic rate (μmol∙m^−2^ s^−1^)			
*A. tanguticus*	11.874 ± 0.336 A, X	12.724 ± 0.519 A, X	14.742 ± 0.485 A, Y
*A. luridus*	9.763 ± 0.465 B, X	17.703 ± 0.431 B, Y	16.676 ± 0.202 B, Y
*A. carniolicoides*	10.792 ± 0.423 AB, X	12.793 ± 0.302 B, Y	14.710 ± 0.510 A, Z
*A. acutangulus*	10.555 ± 0.389 B, X	12.248 ± 0.283 B, Y	12.074 ± 0.293 C, Y
Stomatal conductance (mmol m^−2^ s^−1^)			
*A. tanguticus*	0.152 ± 0.015 A, X	0.243 ± 0.023 A, Y	0.297 ± 0.020 A, Y
*A. luridus*	0.083 ± 0.006 B, X	0.382 ± 0.026 B, Y	0.400 ± 0.029 B, Y
*A. carniolicoides*	0.168 ± 0.008 A, X	0.215 ± 0.010 A, Y	0.270 ± 0.020 AC, Z
*A. acutangulus*	0.168 ± 0.018 A, X	0.208 ± 0.012 A, XY	0.234 ± 0.009 C, Y
Transpiration (mmol m^−2^ s^−1^)			
*A. tanguticus*	4.866 ± 0.366 A, X	6.703 ± 0.447 AB, Y	7.553 ± 0.276 AB, Y
*A. luridus*	3.328 ± 0.232 B, X	9.913 ± 0.356 C, Y	10.026 ± 0.380 C, Y
*A. carniolicoides*	5.165 ± 0.225 A, X	7.184 ± 0.250 B, Y	8.180 ± 0.328 A, Z
*A. acutangulus*	5.055 ± 0.407 A, X	6.048 ± 0.240 A, Y	6.898 ± 0.149 B, Y
Instantaneous water use efficiency			
*A. tanguticus*	2.498 ± 0.164 A, X	1.917 ± 0.059 AB, Y	1.954 ± 0.032 A, Y
*A. luridus*	2.972 ± 0.136 B, X	1.791 ± 0.037 A, Y	1.675 ± 0.063 B, Y
*A. carniolicoides*	2.097 ± 0.065 C, X	1.793 ± 0.079 A, Y	1.817 ± 0.018 C, Z
*A. acutangulus*	2.137 ± 0.140 AC, X	2.039 ± 0.082 B, XY	1.752 ± 0.042 BC, Y
Maximum quantum yield of PS II			
*A. tanguticus*	0.817 ± 0.005 A, X	0.829 ± 0.002 AB, Y	0.823 ± 0.002 A, XY
*A. luridus*	0.830 ± 0.002 B, X	0.834 ± 0.001 A, X	0.831 ± 0.002 B, X
*A. carniolicoides*	0.824 ± 0.002 AB, X	0.823 ± 0.004 B, X	0.829 ± 0.003 B, X
*A. acutangulus*	0.827 ± 0.001 B, X	0.831 ± 0.001 A, Y	0.832 ± 0.001 B, Y
Carbon content			
*A. tanguticus*	0.441 ± 0.006 A, XY	0.431 ± 0.008 A, X	0.455 ± 0.009 A, Y
*A. luridus*	0.424 ± 0.012 A, X	0.413 ± 0.006 AB, X	0.432 ± 0.007 BC, X
*A. carniolicoides*	0.399 ± 0.010 A, X	0.401 ± 0.011 B, X	0.424 ± 0.004 C, X
*A. acutangulus*	0.408 ± 0.029 A, X	0.423 ± 0.008 AB, X	0.437 ± 0.006 AC, X
Nitrogen use efficiency			
*A. tanguticus*	15.038 ± 0.347 A, X	17.924 ± 0.255 A, X	16.794 ± 0.310 A, X
*A. luridus*	16.164 ± 0.494 A, X	17.839 ± 0.482 A, Y	18.115 ± 0.374 AC, Y
*A. carniolicoides*	16.094 ± 0.613 A, X	18.713 ± 0.565 A, Y	18.836 ± 1.019 BC, Y
*A. acutangulus*	17.570 ± 1.980 A, X	17.714 ± 0.466 A, X	16.983 ± 0.373 A, X
Carbon isotope composition			
*A. tanguticus*	−29.583 ± 0.193 A, X	−30.572 ± 0.285 A, Y	−30.823 ± 0.504 A, Y
*A. luridus*	−28.675 ± 0.294 A, X	−30.173 ± 0.235 AB, Y	−30.191 ± 0.527 B, Y
*A. carniolicoides*	−28.630 ± 0.218 A, X	−29.555 ± 0.377 B, Y	−29.401 ± 0.581 C, XY
*A. acutangulus*	−26.793 ± 0.443 B, X	−28.600 ± 0.249 C, Y	−28.848 ± 0.406 C, Y

Values with different letters (X, Y, Z) indicate a significant difference (*P* < 0.05) between water treatments, and those with different letters (A, B, C) indicate a significant difference (*P* < 0.05) between species, based on one-way ANOVA.
