# Supplementary material for: Differentiation in drought tolerance mirrors the geographic distributions of alpine plants on the Qinghai-Tibet Plateau and adjacent highlands
Source: Sci Rep. 2017 Feb 14;7:42466. doi: 10.1038/srep42466 (PMC5307960; doi:10.1038/srep42466)
Supplement: Supplementary Table S1 [file srep42466-s1.pdf]

Differentiation in drought-tolerance mirrors the geographic distributions of alpine plants on the Qinghai-Tibet Plateau and adjacent highlands

Li-Hua Meng, Jie Yang, Wen Guo, Bin Tian, Guang-Jie Chen, Yong-Ping Yang, Yuan-Wen Duan

Supplementary materials

Table S1. Effects of species, water stress treatment and their interaction on all measured variables of seedlings of the four *Anisodus* species, based on two-way ANOVA. *, $P < 0.05$; **, $P < 0.01$; ***, $P < 0.001$.

Variable	Species	Water treatment	Treatment × species interaction
Aboveground biomass	145.37***	61.40***	2.18*
Belowground biomass	12.47***	30.76***	5.91***
Total biomass	110.84***	56.39***	3.06**
Root:shoot ratio	33.64***	0.88	6.36***
Long-term water use efficiency	4.31**	0.09	5.89***
Maximal rate of photosynthesis	30.72***	103.47***	19.018***
Stomatal conductance	12.70***	83.36***	14.12***
Transpiration rate	17.18***	141.17***	18.59***
Instantaneous water use efficiency	4.66**	63.81***	9.43***
Maximum quantum yield of PS II	7.94***	3.89*	1.70
Carbon content	4.46**	3.92*	0.33
Nitrogen use efficiency	1.81	10.36***	1.16
Carbon isotope composition	37.77***	34.19***	1.18
